# ﻿New insights into the mitogenomic phylogeny and evolutionary history of Murinae (Rodentia, Muridae) with the description of a new tribe

**DOI:** 10.3897/zookeys.1233.140676

**Published:** 2025-03-27

**Authors:** Shuang Liu, Songping Zhao, Jing Wang, Changkun Fu, Xuming Wang, Shaoying Liu, Shunde Chen

**Affiliations:** 1 Ministry of Education, Key Laboratory of Land Resources Evaluation and Monitoring in Southwest (Sichuan Normal University), Chengdu, China; 2 College of Life Science, Sichuan Normal University, Chengdu, China3 Sichuan Academy of Forestry, Chengdu, China; 3 Sichuan Academy of Forestry, Chengdu, China

**Keywords:** Divergence analysis, evolutionary history, mitochondrial genome, nocturnal rodents, phylogenetic analysis, taxonomy, tribal classification, *Vernaya*

## Abstract

Murinae is the largest known subfamily of Muridae and includes 15 tribes and 3 genera (*incertae sedis*). Although the phylogeny of Murinae has been studied, its phylogenetic relationships have not been completely elucidated. We used phylogenetic framework and molecular dating methodologies with the vast majority of available mitochondrial genomes to disentangle the phylogenetic relationships and evolutionary history of Murinae. Sixteen tribes were identified within the Murinae subfamily. Among these, fifteen tribes were found to be consistent with those currently recognized. Hapalomyini (Clade A) was located at the base of the Murinae clade with strong nodal support contrary to previous studies, which showed that Phloeomyini diverged first. The Clade B consisted of Micromyini, Rattini, and the genus *Vernaya*. *Vernaya* cannot be accommodated in any existing tribe. The origin of Murinae dates back to 17.22 Ma. The split between Micromyini and Vernayini was dated to 11.69 Ma during the Miocene, indicating that they were both early branches of Murinae. Combined with the differences between *Vernaya* and its sister tribes (Micromyini and Rattini) in morphology, skull and teeth, we validated a new tribe, Vernayini**tribe nov.** We believe that it is necessary to combine morphological and molecular perspectives (especially from a genome-wide perspective) to determine the phylogenetic position of tribes with an uncertain taxonomic position in Murinae.

## ﻿Introduction

Murinae comprises 135 genera and 656 species and is the largest subfamily of Muridae (Wilson and Mittermeier 2018). Muridae exhibit a greater degree of variation in their diversification rates compared to any other rodent group (Fabre et al. 2012). In addition, owing to the medical importance and genomic benefits of Murinae species, sequencing, phylogeny, and divergence analyses are particularly important. The phylogenetic relationships of some subfamilies have been explained from various aspects; however, those of Murinae have not been fully elucidated (Steppan et al. 2005). To fill this void in knowledge, Steppan et al. (2005) clarified the phylogeny of Murinae, which consisted of seven distinct geographic lineages. They achieved this by employing three nuclear genes and one mitochondrial fragment and inferred that Murinae originated in Southeast Asia. Musser and Carleton (2005) divided Murinae into 29 divisions. Lecompte et al. (2008) proposed describing the phylogenetic relationships of Murinae at the tribe level and pointed out that Phloeomyini is basal to Murinae. Fabre et al. (2013) deduced the phylogenetic relationships of Murinae and reconstructed the biogeographic history, also showing that Phloeomyini is located at the base of Murinae; they found that *Micromys* belongs to Murini, rather than a separate tribe, and that Murinae has nine tribes. Meschersky et al. (2016) determined that *Chiropodomys* is neither a member of the *Micromys* division nor is it closely related to *Hapalomys*. Missoup et al. (2016) clarified the phylogenetic relationships among Arvicanthini species. In the same year, Pagès et al. (2016) used molecular analysis and morphological comparison methods to determine the taxonomical status of the *Micromys* and *Pithecheir* divisions in Murinae. According to recent research, Murinae is divided into 15 tribes, and 3 genera with an undetermined status (Wilson and Mittermeier 2018). This suggests that many genera have uncertain taxonomic status in Murinae, which are named Murinae*incertae sedis* (Pagès et al. 2016).

*Vernaya* belongs to the subfamily Murinae and is regarded as a monotypic genus containing only one species, *Vernayafulva*. Zhao et al. (2023) performed molecular and morphological analyses on this taxon, and determined that *Vernaya* is not a monotypic genus. Instead, it contains four species: *Vernayafulva* (Allen, 1927), *Vernayaforamena* Wang, Hu & Chen, 1980, *Vernayanushanensis* Zhao, Liu, Jiang, Liu & Chen, 2023, and *Vernayameiguites* Zhao, Li, Wang, Jiang, Liu & Chen, 2023. Thus, the phylogeny of the genus in Murinae remains unknown.

Mitochondrial genomes have been used in many studies to explore the phylogeny of species (Rasmussen and Arnason 1999; Kuang et al. 2019; Song et al. 2022; Huo et al. 2023). However, based on their genomes, no studies have investigated the phylogeny of Murinae. To determine the phylogenetic relationships of Murinae and the taxonomic status of *Vernaya* in Murinae, we conducted whole mitochondrial genome sequencing for one individual of each of the four species of the genus, constructed a phylogenetic tree of Murinae, and performed a divergence time calculation to determine the evolutionary history.

## ﻿Material and methods

### ﻿Specimen collection, DNA extraction and sequencing, assembly and annotation

In the present study, we sequenced one individual of each of the four species in *Vernaya*. The four specimens were collected using snap traps from Yunnan and Sichuan, China (we sequenced the mitochondrial genomes (mtGenomes) of the first four species in Table 1). All individuals in this table were used for morphological comparative analysis later.

**Table 1.** Collection information of specimens.

Species	Field ID	Museum number	Locality
*Vernayafulva*	csd4405	SCNU02747	Lanping, Yunnan
*Vernayaforamena*	csd3540	SAF201518	Pingwu, Sichuan
*Vernayanushanensis*	csd3561	SAF19287	Yunlong, Yunnan
*Vernayameiguites*	csd3537	SAF201653	Meigu, Sichuan
*Micromysminutus*	csd1525	SAF19383	Habahe, Xinjiang
*Rattustanezumi*	XZ021	SCNU00173	Yadong, Xizang

The implementation of trapping across all locations included a broad utilization of various trap models, such as Victor snap traps, Museum Special traps, and Sherman live traps. The process of capturing the specimens was executed with the aid of cage-style traps. Once captured, the small mammals were transported to the lab, where they underwent bloodletting under the influence of isoflurane anesthesia, administered on a heated mat to ensure their comfort and to reduce any potential suffering. Fresh liver or muscle was obtained and preserved in anhydrous ethanol in the field, and stored in a −80 °C freezer upon return to the laboratory. Tissues and specimens were stored at the Sichuan Academy of Forestry Sciences (SAF) and Sichuan Normal University (SCNU). All fieldwork complied with legal regulations in China, and sampling was carried out following local legislation. This study was approved by and conducted according to the guidelines of the Animal Ethics Committee of Sichuan Normal University.

We used the animal tissue DNA extraction kit of Chengdu Foregene Company Limited to extract DNA following the manufacturer’s instructions, and then sent it to Novogene (Beijing, China) for high-throughput sequencing using the Illumina NovoSeq 6000. The original data were spliced using MitoZ in the Linux system with reference to the whole-genome sequence of *Vernaya* species (Meng et al. 2019). The Mitos database (http://mitos.bioinf.uni-leipzig.de/index.py) was used to preliminary annotate the mtGenomes and to detect the sequences of protein-coding genes, rRNA, and tRNA (Bernt et al. 2013). The sequences were annotated using CGView (https://proksee.ca/) to obtain a structural map of the mitochondrial genome (Grant et al. 2023).

### ﻿Phylogenetic analysis of Murinae

In the present study, based on the mtGenomes of four species of *Vernaya*, the taxonomic status of the genus was explored. According to the latest classification relationship of Murinae, we downloaded the mtGenome sequences of some Murinae species from NCBI (https://www.ncbi.nlm.nih.gov/), based on the classification of the tribes of Murinae (except for three tribes) (Wilson and Mittermeier 2018). For species without available mtGenome sequences, *Cyt b* gene sequences were obtained from NCBI (Table 2).

**Table 2.** Accession numbers of mtGenomes and *Cyt b* sequences of the species of Murinae.

Tribes	Species	Complete mitochondrial sequence	*Cyt b* gene
Outgroups	*Merionestamariscinus*	NC034314	
	*Merionesmeridianus*	NC027684	
Vernayini	*Vernayaforamena*	OR085220	
	*Vernayafulva*	OR085222	
	*Vernayameiguites*	OR085219	
	*Vernayanushanensis*	OR085221	
Malacomyini	*Malacomysedwardsi*	MN964121	
Rattini	*Bandicotaindica*	KT029807	
	*Niviventerandersoni*	NC060500	
	*Rattusrattus*	NC012374	
	*Chiromyscuslangbianis*	NC084241	
	*Leopoldamyssabanus*	MT259591	
	*Leopoldamysneilli*	JX573334	
	*Maxomyssurifer*	NC036732	
	*Margaretamysparvus*		MN273044
Micromyini	*Micromysminutus*	NC027932	AB201996
	*Micromyserythrotis*	MW389539	
Praomyini	*Stenocephalemysalbipes*	NC051514	
	*Heimyscusfumosus*	NC049120	
	*Hylomyscusdenniae*	MN845743	
	*Mastomyscoucha*	NC036018	
	*Praomysrostratus*	NC049115	
Millardiini	*Millardiameltada*	MN807616	
Chiropodomyini	*Chiropodomysgliroides*	NC049121	KJ772301
	*Chiropodomysgliroides*	MN964124	
Hydromyini	*Baiyankamyshabbema*		MN273033
	*Melomysburtoni*	NC049118	
	*Xeromysmyoides*		EU349790
	*Leggadinalakedownensis*	NC014696	
	*Pseudomyschapmani*	NC014698	
Vandeleurini	*Vandeleuriaoleracea*		KY754177
Apodemini	*Apodemusdraco*	HQ333255	
	*Apodemuslatronum*	HQ333256	
	*Tokudaiaosimensis*	LC778283	
	*Tokudaiaosimensis*		AB033703
Pithecheirini	*Pithecheirparvus*		MG189672
Otomyini	*Otomyssungae*		JF795993
	*Otomyszinki*		JF795989
	*Otomystypus*	NC053811	
	*Otomysirroratus*	MK166028	
	*Parotomysbrantsii*		KY754096
Arvicanthini	*Golundaellioti*	NC053815	MN807614
	*Golundaellioti*		KY986802
	*Desmomysharringtoni*	MN807595	MT084863
	*Arvicanthisnairobae*		MK239825
Arvicanthini	*Arvicanthissomalicus*	NC053801	
	*Dephomysdefua*	NC053808	MF992073
	*Hybomyslunaris*		MF680490
	*Hybomystrivirgatus*	NC053810	
	*Rhabdomyspumilio*		AF533116
	*Thallomyspaedulcus*		KU724036
Hapalomyini	*Hapalomysdelacouri*	MZ159976	MG189666
Phloeomyini	*Phloeomyscumingi*		MH330620
	*Batomysgranti*		EU349738
	*Crateromysschadenbergi*		MH330619
Murini	*Musspretus*	OR840825	
	*Musmusculus*	LC644162	KF839627

We chose *Merionestamariscinus* Pallas, 1773 and *Merionesmeridianus* Pallas, 1773 as outgroups and downloaded their complete mitogenomes from the NCBI. The sequences of Murinae species obtained from NCBI and the sequences of four individuals of *Vernaya* were imported into MEGA 5 software. The gene sequences were compared; inconsistent or uncertain sequences were manually corrected and removed, and the aligned FASTA file was exported. We applied Bayesian inference (BI) and maximum-likelihood (ML) methods to infer the phylogenetic relationships. BI analyses were performed using BEAST v. 1.7 (Drummond et al. 2012). First, jModelTest v. 2.1.7 was used to calculate the optimal model, GTR+G (Posada and Manousiouthakis 2008). BEAUti was used to set the following parameters: model calculation involved selecting an inhomogeneous model gamma, an alternative model GTR, a relaxed molecular clock, and the Yule process; the search chain was run for 100 million generations and sampled every 5000 generations; and the remaining parameters were set to default. We then used BEAST v. 1.7 to construct a phylogenetic tree. We employed Tracer v. 1.6 to verify that the effective sample sizes (ESSs) exceeded 200; therefore, the result tended to be reliable (Rambaut and Drummond 2013). Burn-in was discarded via TreeAnnotator v. 1.6.1. ML analyses were conducted using W-IQ-TREE (http://iqtree.cibiv.univie.ac.at) as described by Trifinopoulos et al. (2016). The analyses used the rapid bootstrapping algorithm with 1000 replicates. The final BI tree and ML tree were decorated and embellished using FigTree v. 1.4.3, and the posterior probability and bootstrap values of each branch were displayed (Rambaut 2016). We also evaluated the phylogeny of Murinae using BI and ML methods based on the complete mitochondrial sequences and removed three tribes containing only *Cyt b* sequences to locate the *Vernaya* genus. The sequences used are shown in Suppl. material 1: table S1. The process of all phylogenetic tree building and annotation steps followed the above protocol.

### ﻿Estimating dates of divergence

We estimated the divergence time by using two types of sequences. For species that do not have mtGenome sequences, we utilized *Cyt b* sequences. For other species, we employed mtGenome sequences from some species belonging to 15 tribes within Murinae. Additionally, we used mtGenome sequences from one individual of each of the four selected species of *Vernaya*. Sequence selection was the same as that for the phylogenetic tree. Data were analyzed using BEAST v. 1.7. Divergence times were estimated using five fossil-based calibration intervals as described by Pagès et al. (2016) and Aghová et al. (2018). We used the following constraints: (1) The stem Apodemini fossils (11 Ma min.) from the Early Vallesian were used to constrain the split between Apodemini/Millardiini (Most Recent Common Ancestor (MRCA) of *Apodemus*/*Tokudaia*) and Praomyini/Murini (MRCA of *Mus*/*Praomys*/*Mastomys* Clade) (upper 95%: 8.91–21.8 Ma) (Suárez and Mein 1998; Vangengeim et al. 2006); (2) The first fossil record of *Mus* (*Musauctor*) was used to represent the minimum divergence at 5.7 Ma (upper 95%: 4.66–11.07 Ma) between different *Mus* lineages (*Musmusculus*/*Muspahari*/*Mussetulosus*) (Jacobs et al. 1990; Jacobs and Downs 1994; Lundrigan et al. 2002); (3) We used the African crown Arvicanthini lineage from the Late Miocene (median age 6 Ma; from the Tortonian) and a soft maximum prior extending to the Serravalian (upper 95%: 3.91–16.81 Ma) as a constraint of the MRCA of Arvicanthini (Winkler 2002); (4) We set a minimum constraint for the MRCA of Hydromyini, using the first Australian fossil evidence dated at 3.4 Ma (upper 95%: 1.3–14.21 Ma); and (5) The divergence time of Murinae (15.9 Ma, upper 95%:14.06–18.15 Ma) was used as calibration point in the present analyses (Aghová et al. 2018).

All fossil dating age constraints are considered lognormal distributions (Tedford et al. 1992; Ho 2007; Rowe et al. 2008; López-Antoñanzas et al. 2024). The best-fitting substitution models for each partition were selected using the jModelTest results. A general time-reversible model was used as the substitution model. The Yule process of speciation was selected as the tree prior and combined with a relaxed lognormal molecular clock model. Each analysis was performed for 100 million generations, with samples collected every 5000 generations. The posterior distribution and ESS for parameters greater than 200 were calculated using Tracer v. 1.6. TreeAnnotator v. 1.6.1, which was set to the top 10% of the generation, was used to determine the necessary burn-in portion. We then viewed and identified the divergence tree in FigTree v. 1.4.3.

### ﻿Abbreviations

**mtGenome**: mitochondrial genome; **MRCA**: The most recent common ancestor; **QTP**: Qinghai-Tibetan Plateau; **Ma**: Megaannus; *V.foramena*: *Vernayaforamena*; *V.f.foramena*: *Vernayaforamenaforamena*; *V.fulva*; *Vernayafulva*; *V.meiguites*: *Vernayameiguites*; *V.nushanensis*: *Vernayanushanensis*; **SCNU**: Sichuan Normal University; SAF: Sichuan Academy of Forestry Sciences.

## ﻿Results

### ﻿Characteristics of the mtGenomes in *Vernaya*

**Figure 1.**
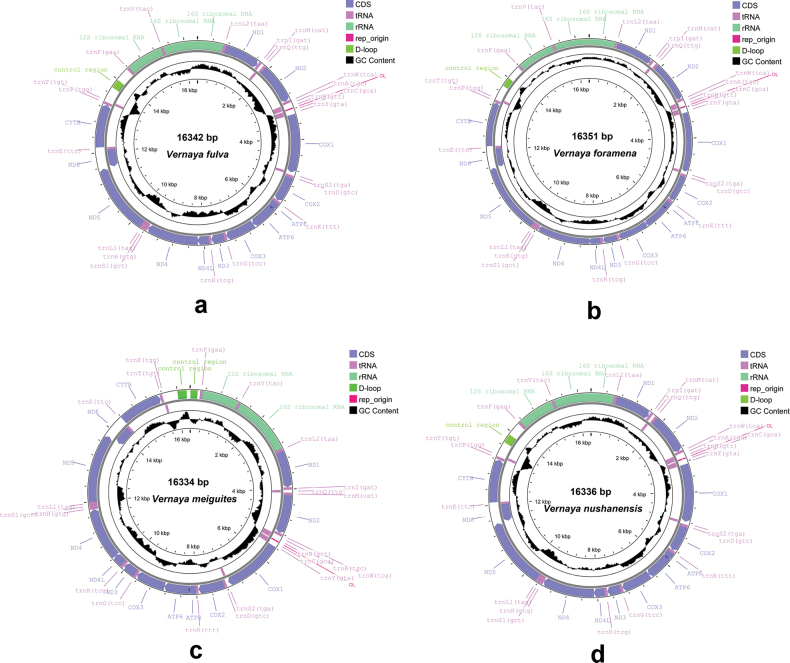
MtGenomes structure map of the species of *Vernaya***a***Vernayafulva* (SCNU02747) **b***Vernayaforamena* (SAF201518) **c***Vernayameiguites* (SAF201613) **d***Vernayanushanensis* (SAF19287)).

The structure of mtGenomes exhibit a striking resemblance to that found in typical vertebrates and other rodents. It comprises 13 protein-coding genes, 2 rRNA genes, 22 tRNA genes, and 1 control region in *V.fulva* (Fig. 1a), *V.foramena* (Fig. 1b), and *V.nushanensis* (Fig. 1d), and two control regions (two D-loop) in *V.meiguites* (Fig. 1c), which is not common in mammals, including rodents. The size of the mitochondrial genome is 16,334 bp in *V.meiguites* to 16,351 bp in *V.foramena*. While ND6 and eight tRNA genes reside on the light strand, the remaining mtGenome genes (such as PCGs, rRNAs, and other tRNAs) along with the control region, are located on the heavy strand (Fig. 1a–d). All *Vernaya* mtGenomes showed highly similar nucleotide composition biases. All mtGenomes were AT-rich, with AT content ranging from 65.38% (*V.fulva* and *V.foramena*) to 65.78% (*V.meiguites*), suggesting strand heterogeneity in the nucleotide composition.

### ﻿Phylogenetics and divergence in Murinae based on mtGenomes

**Figure 2.**
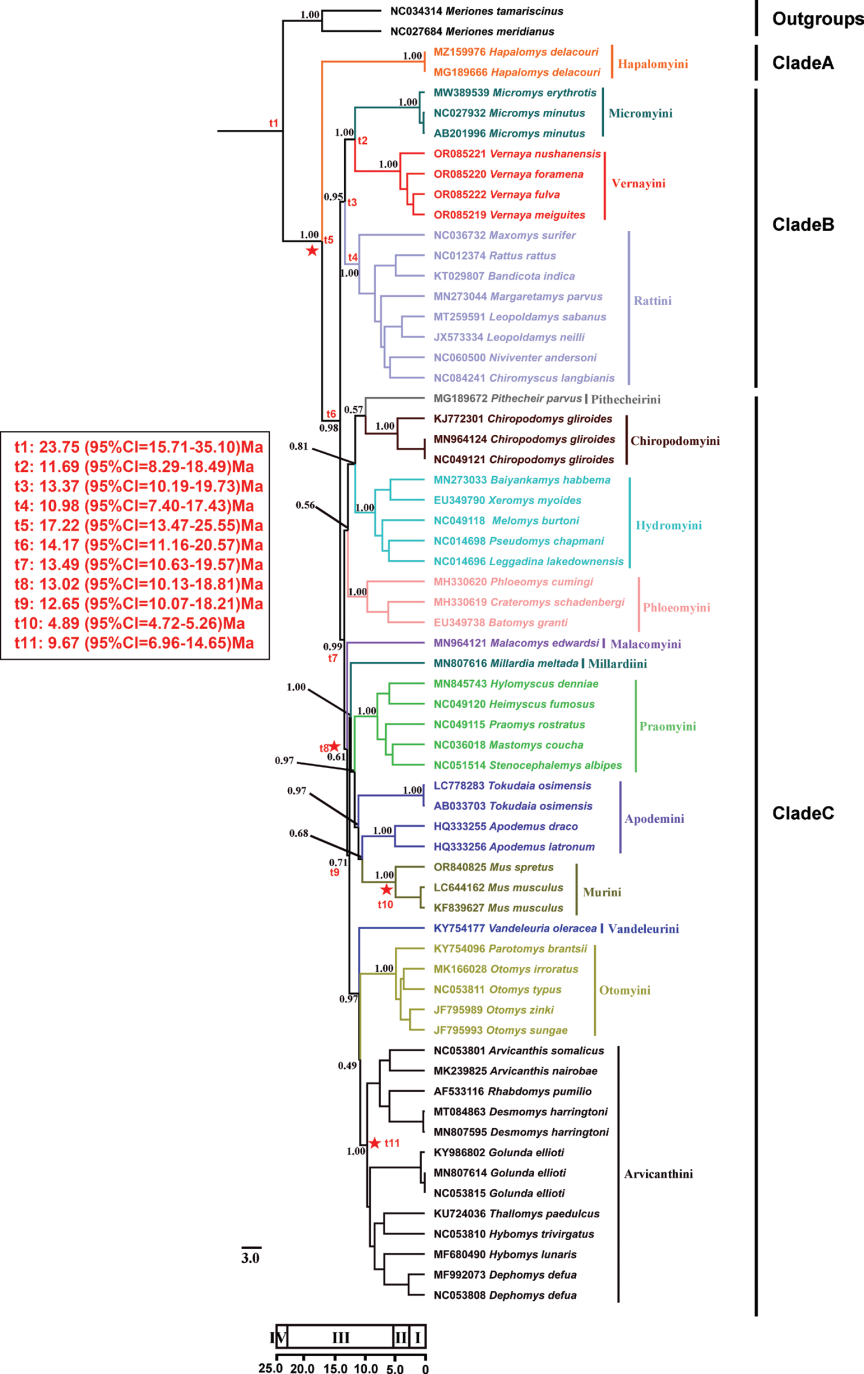
Phylogenetic and molecular dating results for Murinae and close-relative lineages. The tree is a chronogram (uncorrelated log-normal molecular clock) based on a BEAST MCMC analysis of the mtGenome sequences (except for three tribes). We propose elevating the genus *Vernaya* to tribe Vernayini. Clocks indicate the fossil calibration points used for molecular dating (referring to Pagès et al. (2016) and Aghová et al. (2018)). Red stars indicate calibration points. The values at the nodes are posterior probabilities (PP) obtained by Bayesian analysis. t1-t11 represent the divergence time of some important nodes. NCBI accession numbers for each species are shown on the branch; different colors of the branch and taxa represent different tribes. Numbers represent: Ⅰ. Quaternary; Ⅱ. Pliocene; Ⅲ. Miocene; Ⅳ. Oligocene.

**Figure 3.**
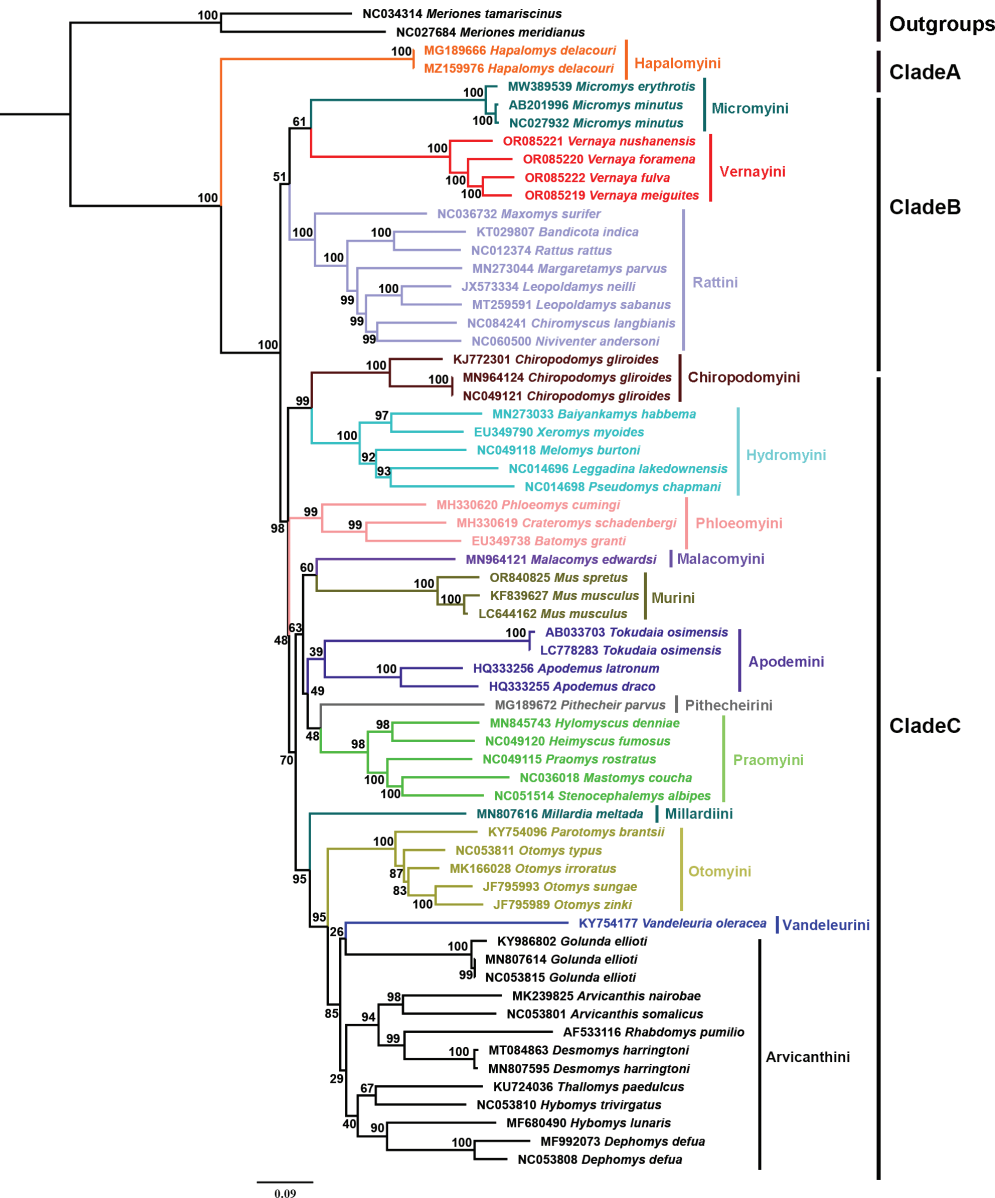
ML tree for Murinae and close-relative lineages based on the mtGenome sequences. 1000 bootstrap replicates were applied. Numbers at nodes represent ML bootstrap support values.

We constructed a BI phylogenetic tree and a ML tree based on the mtGenomes of four species of *Vernaya* and the mtGenome sequences of the other Murinae species, except for those of three tribes (Fig. 2 for BI tree and Fig. 3 for ML tree). The accession numbers of the sequences are listed in Table 2.

Three clades and sixteen tribes were retrieved from Murinae in our study, and fifteen of them correspond to currently recognized tribes (Figs 2, 3).

In both phylogenetic trees, the positions of Clade A, Clade B, and Clade C within Murinae were roughly the same. Hapalomyini (Clade A), located at the base of Murinae, was the first to differentiate and was strongly supported (PP = 1.00, BP = 100), with the assumptions proposed in previous research (Badenhorst et al. 2012; Meschersky et al. 2016; Pagès et al. 2016). Clade B consisted of Micromyini, Rattini, and *Vernaya* (PP = 0.95), all of which were grouped together and were well supported (PP = 1.00, BP = 100). The *Vernaya* genus cannot be accommodated in any existing tribe. Interestingly, it was sister to Micromyini, and combined to form a sister group to Rattini (Figs 2, 3, Suppl. material 1: fig. S1). In both trees, the remaining 12 tribes constituted a large branch (Clade C). However, there were numerous disparities in this branch between the BI tree and ML tree. For example, our ML tree grouped Chiropodomyini together with Hydromyini (BP = 99), while our BI tree placed Pithecheirini as sister taxa to the Chiropodomyini tribe but with weak support (PP = 0.57). Since the location of *Vernaya* within Murinae is the same in both trees, we shall mainly discuss the BI tree.

In the BI tree, Clade C was composed of four smaller clades. One of these smaller clades was made up of Phloeomyini, Pithecheirini, Chiropodomyini, and Hydromyini. Phloeomyini was positioned at the base of this particular clade. But in some previous studies, Phloeomyini was located at the base of Murinae (Steppan et al. 2005; Lecompte et al. 2008; Fabre et al. 2013; Schenk et al. 2013; Missoup et al. 2016). However, Chiropodomyini was sister to Pithecheirini, which was different from a previous study (Pagès et al. 2016). Malacomyini was the first to diverge from the large clade that comprised Malacomyini, Millardiini, Praomyini, Apodemini, Murini, Vandeleurini, Otomyini, and Arvicanthini. The clade consisting of Millardiini, Praomyini, Apodemini, and Murini was a sister to the clade consisting of Vandeleurini, Otomyini, and Arvicanthini. Within the clade consisting of Millardiini, Praomyini, Apodemini, and Murini, Murini was sister to Apodemini, and they formed a sister group with Praomyini, and then formed a sister group with Millardiini. However, in previous studies, Murini was sister to Praomyini and the clade combining the two tribes was sister to Apodemini (Steppan et al. 2005; Lecompte et al. 2008; Fabre et al. 2013; Schenk et al. 2013; Pagès et al. 2016). In the clade consisting of Vandeleurini, Otomyini and Arvicanthini, Vandeleurini diverged first, followed by Otomyini and Arvicanthini. Otomyini and Arvicanthini were sister groups, which was similar to previous studies (Fabre et al. 2013; Schenk et al. 2013; Missoup et al. 2016; Pagès et al. 2016).

The estimated divergence time is shown in the BI topology in Fig. 2. The results showed that the divergence between Gerbillinae and Murinae is estimated to have occurred during the Oligocene (23.75 Ma, 95% CI = 15.71–35.10). The MRCA of Murinae can be traced back to the Miocene (17.22 Ma, 95% CI = 13.47–25.55), which was also the time when Hapalomyini first diverged from Murinae. Cladogenesis between Clade B and Clade C was dated to 14.17 Ma (95% CI = 11.16–20.57 Ma). The divergence between the clade consisting of Phloeomyini, Pithecheirini, Chiropodomyini, and Hydromyini and the large clade consisting of the other tribes in Clade C was dated to 13.49 Ma (95% CI = 10.63–19.57 Ma). In Clade B, the divergence between Rattini and the other two groups (Micromyini and *Vernaya*) was estimated to have occurred around 13.37 Ma (95% CI = 10.19–19.73 Ma). The MRCA of this tribe was calculated to have occurred in the Miocene, approximately 10.98 Ma (95% CI = 7.40–17.43 Ma). The split between Micromyini and *Vernaya* dates to 11.69 Ma (95% CI = 8.29–18.49 Ma) during the Miocene. This chronology, along with the MRCA chronology of Micromyini, indicates an early branch of both *Vernaya* and Micromyini of Murinae.

## ﻿Discussion

The higher-level classification of Murinae remains controversial. Fabre et al. (2015) summarized the progress of rodent taxonomy, but retained the “division” arrangement of the rodent subfamily instead of the tribe arrangement. Pagès et al. (2016) re-examined the *Micromys* and *Pithecheir* divisions using molecular and morphological evidence to demonstrate the multilineage status. The current classification of Murinae includes 15 tribes (Wilson and Mittermeier 2018). Previous studies have obtained different phylogenetic trees of Murinae based on different gene segments; however, the locations of some genera or species in Murinae have not been resolved. Therefore, the taxonomic classification of some tribes with an uncertain taxonomic status should be elucidated (Pagès et al. 2016). Our research results showed that we need to further sample, sequence, analyze and study the tribes in Clade C.

**Figure 4.**
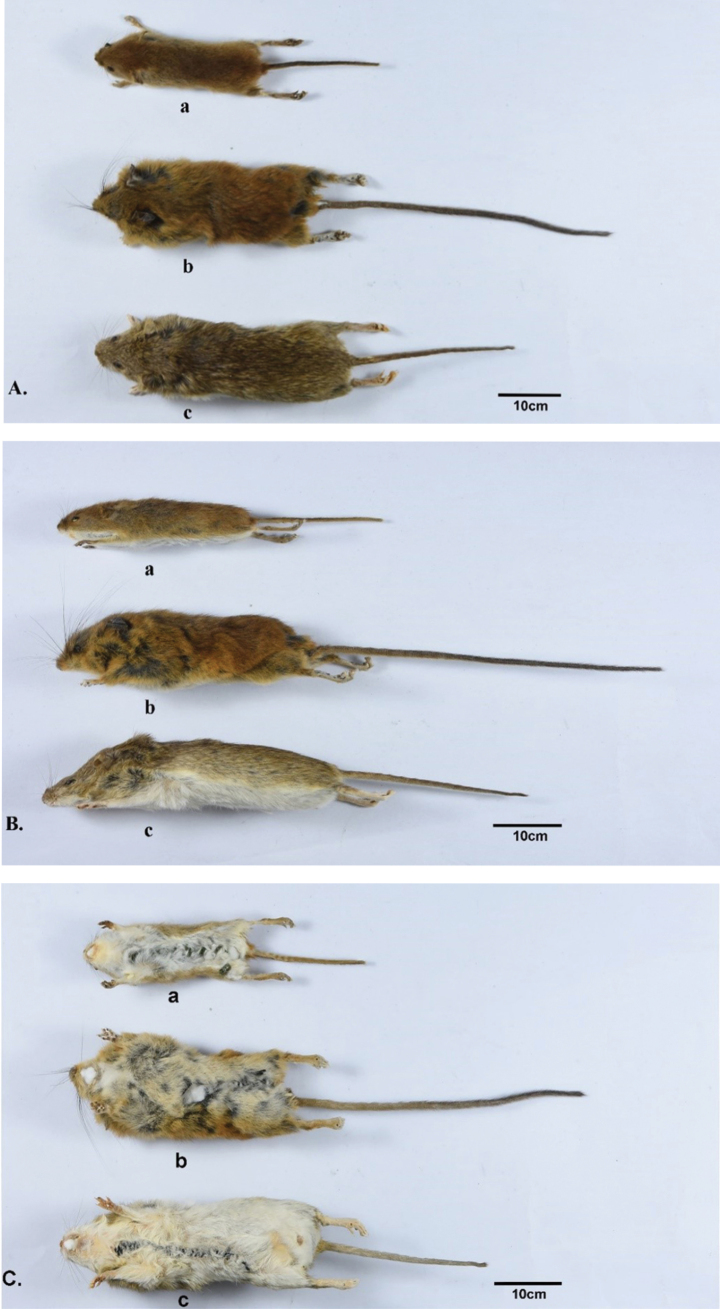
Skin specimens from three different genera in dorsal (**A**), lateral (**B**), and ventral (**C**) view. a is a skin specimen of *Micromysminutus* (SAF19383); b is a skin specimen of *Vernayafulva* (SCNU02747); c is a skin specimen of *Rattustanezumi* (SCNU00173).

The teeth and outline of the tribes confirm our molecular results and a review of a new tribe. Through the construction of phylogenetic trees, we confirmed the monophyly of *Vernaya* within Murinae, with strong nodal support (Fig. 2, PP = 1.00; Fig. 3, BP = 100; Suppl. material 1: fig. S1, PP = 1.00; Suppl. material 1: fig. S2, BP = 100); it is designated as an independent tribe in Murinae. In both the ML and BI trees (Figs 2, 3), *Vernaya* formed a clade (Clade B) with Micromyini and Rattini, suggesting close phylogenetic relationships among these groups. The morphology, skull, and teeth are signature features of Murinae (Carleton and Musser 1984). Numerous studies have erected novel taxa based on the disparities in these characteristics (Pu et al. 2022; Chen et al. 2024). Specimens of *Vernaya* and its sister groups were examined. *Vernaya* had a larger head-to-body length ratio than *Micromys* and *Rattus* (Fig. 4A–C).

**Figure 5.**
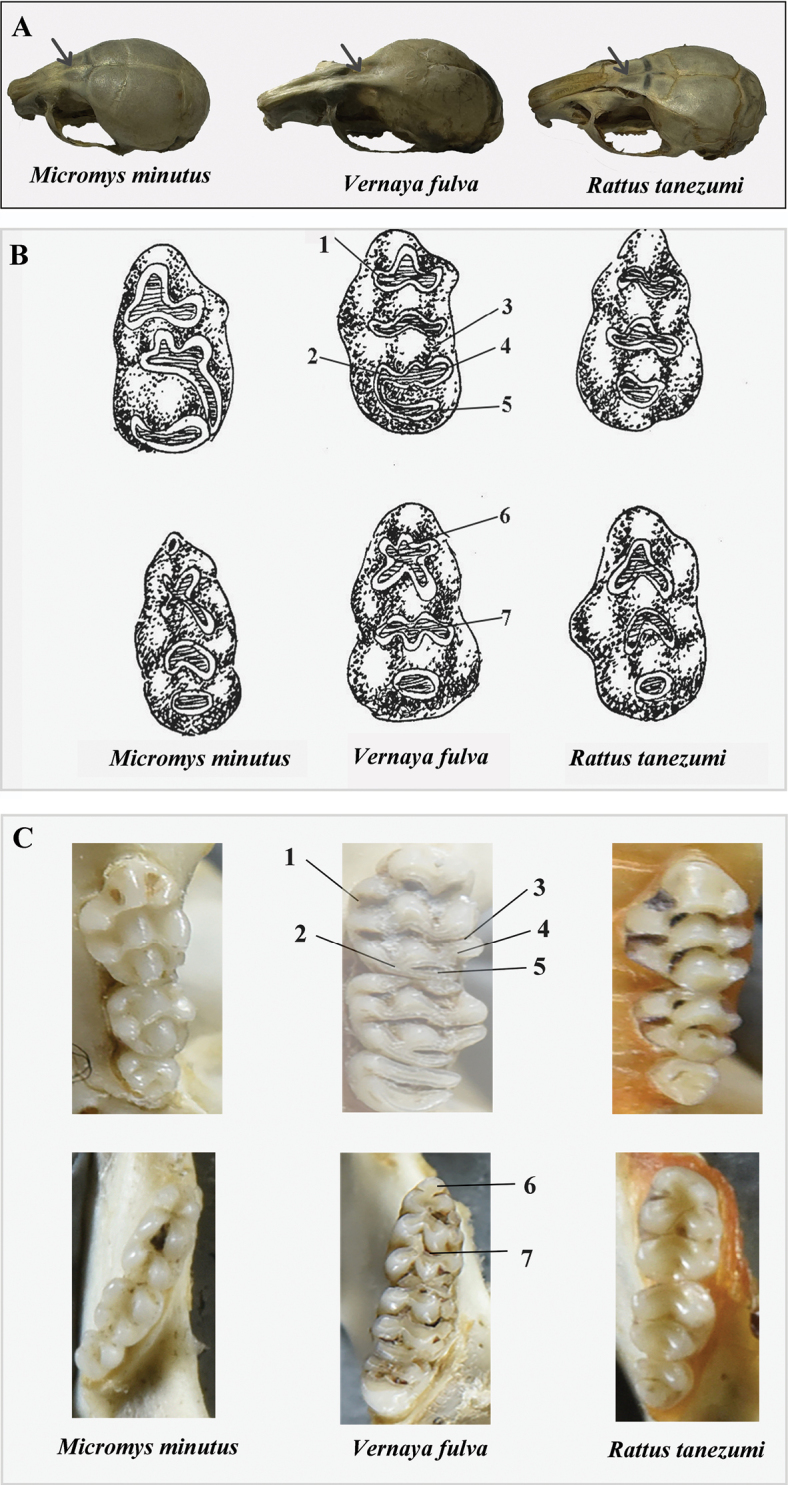
Comparison of skull and teeth morphology. Based on the results of phylogenetic trees, the skull and molar morphology of species in *Vernaya* were compared with that of the species in the genera closely related in the phylogenetic tree (*Micromys* and *Rattus*) **A** comparison of skull morphology. Arrows indicate the presence or absence of longitudinal depressions in the interorbital region of the skull **B** comparison of morphology of the first molar. The 6 images are hand-drawn pictures of the molars M^1^ (upper 3 images) and m_1_ (lower 3 images). The numbers in the figure correspond to those in Table 3**C** comparison of the morphology of the molars. The 6 images are physical images of the upper molars (upper 3 images) and lower molars (lower 3 images). The numbers in the figure correspond to those in the Table 3. *Micromysminutus* (SAF19383); *Vernayafulva* (SCNU02747); *Rattustanezumi* (SCNU00173).

Moreover, there were longitudinal depressions in the interorbital region of the skull in *Vernaya* (Smith and Xie 2008). However, this was absent in *Micromys* and *Rattus* (Fig. 5A). We examined the molars of three genera: *Vernaya*, *Micromys*, and *Rattus*. Revising the molar traits of the species in light of our novel molecular classification resulted in an overall consistent picture (Fig. 5B, C). The dental morphology of *Vernaya* displays characteristics that fall within the broad spectrum of morphological diversity displayed by Micromyini and Rattini. In Micromyini, the presence of an obvious odontoid on the first transverse ridge of M^1^ is a characteristic shared by both Vernayini and Micromyini. In Rattini, the separation of the paracone and metacone on the second and third transverse ridges of M^1^ in Vernayini is similar to the separation of the paracone and metacone seen in Rattini (Fig. 5B, C). In addition, Table 3 also shows some similarities between the *Vernaya* genus and the other two tribes. However, M^3^ of *Vernaya* has two tabular transverse ridges that are not found in Micromyini or Rattini. Additionally, some dissimilarities were observed between the upper and lower molars (Fig. 5C).

**Table 3.** Dental characters of genus *Vernaya* compared with its sister taxa. The morphological comparison in light of the molecular phylogeny obtained in this study. Refer to Pagès et al. (2016) for the naming of each part.

	Corresponding number in Fig. 5B, C		*Micromysminutus*	*Vernayafulva*	*Rattustanezumi*
M^1^	1	First transverse ridge of M^1^	Obvious odontoid	Obvious odontoid	Unobvious odontoid
	2	t7	Present	Present	Absent
	3	Second and third transverse ridge of M^1^	Paracone-metacone junction	Paracone-metacone separation	Paracone-metacone separation
	4	t9	Degeneration	Large and no degradation	Large and no degradation
	5	Third transverse ridge of M^1^	Posteroloph absent	Posteroloph present	Posteroloph absent
m_1_	6	tma and the first transverse ridge of m_1_	Separated	Connected and protruding	Connected but not prominent
	7	Second transverse ridge of m_1_	3 posteroloph	2 posteroloph	2 posteroloph

A character comparison between M^1^ and m_1_ of *Vernaya* and its sister taxon is presented in Table 3 and Fig. 5B, C. The dental morphology of *Vernaya* strongly differs from that of Rattini and Micromyini in its posteroloph of the third transverse ridge of M^1^ ((5) in Fig. 5B, C), and its tma and the first transverse ridge of m_1_ are connected and protruding ((6) in Fig. 5B, C).

This level of dental dissimilarity supports the phylogenetic position of these tribes. Our molecular phylogenetic analyses indicated that *Vernaya* cannot be accommodated in any of the existing tribes. This is supported by the morphological data, which provide strong support for *Vernaya* from related taxa (*Micromys* and *Rattus*). Accordingly, a new tribe is warranted and is described below as tribe Vernayini, following the naming of tribes by Lecompte et al. (2008).

### ﻿Systematics

**Order: RODENTIA Bowdich, 1821**

**Suborder: Myomorpha Brandt, 1855**

**Superfamily: Muroidea Illiger, 1811**

**Family: Muridae Illiger, 1811**

**Subfamily: Murinae Illiger, 1811**

#### ﻿ Vernayini

Taxon classificationAnimaliaRodentiaMuridae

Liu, Zhao, Liu & Chen, tribe nov.

D5B51E38-3D52-55BD-9628-9830C8177509

https://zoobank.org/89A47D54-870B-45CE-B5C5-EA463CC63019

##### Etymology.

The tribal name is formed by adding to the stem of the name of the type genus *Vernaya*, the suffix ini; thus, the name of the tribe becomes Vernay + ini = Vernayini.

##### Type genus.

*Vernaya* Anthony, 1941.

##### Genera included.

The single genus *Vernaya* comprises four species: *Vernayafulva* (Allen, 1927), *Vernayaforamena* Wang, Hu & Chen, 1980, *Vernayameiguites* Zhao, Li, Wang, Jiang, Liu & Chen, 2023 and *Vernayanushanensis* Zhao, Liu, Jiang, Liu & Chen, 2023.

##### Diagnosis.

Vernayini is a tribe of small, arboreal, nocturnal rodents within the subfamily Murinae, which are well-adapted for climbing. These rodents are characterized by their medium-sized bodies, long, soft fur, and a unique combination of morphological features that distinguish them from other rodent groups. The hindfoot is less than 25 mm in length, and the total skull length is under 35 mm. Notably, the tail is particularly long, approximately twice the length of the head and body combined, and it is covered with fine scales, remaining hairless. Both the fifth digit of the forefoot and the first digit of the hindfoot are equipped with flattened nails, with the thumb capable of opposing the other fingers. The first digit of the hindfoot is semi-opposed and has a flattened nail instead of a claw, and both the fifth finger and the fifth toe have one claw each. The skull of Vernayini rodents features a longitudinal depression in the interorbital region, which also frequently contains two unossified sockets. The molar morphology of this tribe is quite distinctive, featuring an incisive foramen that extends backward to the front of the first molar. The third molar, M^3^, is notable for its two tabular transverse ridges. Furthermore, the posterior lobe of the third transverse ridge on the first molar, M^1^, is significantly different from what is seen in Rattini and Micromyini. Members of this tribe are skilled climbers, frequently active on large trees and plants like bananas. Their activity peaks during the morning, and their diet includes plant fruits, seeds, and insects (Pan et al. 2007; Smith and Xie 2008; Wei et al. 2022).

##### Distribution.

Vernayini is primarily found in the mountainous regions of southwestern China, extending into northern Myanmar. The four species within this tribe have distinct distribution patterns (Zhao et al. 2023), as follows:

*Vernayafulva*: found in western Yunnan Province, China, west of the Lancang River, and extends into northern Myanmar.

*Vernayaforamena*: distributed in the Qinling Mountains of northern Sichuan, southern Gansu, southwestern Shaanxi, central Sichuan, the Qionglai Mountain District in western Sichuan, and northeastern Chongqing.

*Vernayameiguites*: distributed in the Meigu Dafengding National Nature Reserve, Mabian Dafengding National Nature Reserve, and adjacent areas, as well as the Gongga Mountains in the middle of the Hengduan Mountains.

*Vernayanushanensis*: found in Xuemeng Mountain, Lushui, Caojian, Dali, Yunnan Province, China, likely with the Lancang River (upper Mekong River in China) as its eastern boundary.

The divergence of arboreal animals may be related to climatic and geological movements. Both *Vernaya* and *Micromys* are climbing species. *Micromys* is found in various regions of the world, including Myanmar, India, Vietnam, and Russia. In China, its distribution extends to Sichuan, Chongqing, Yunnan, and the southern part of Shaanxi (Wei et al. 2022). This distribution overlaps with the distribution of *Vernaya* species. According to the divergence time results, we noticed that the divergence of *Micromys* and *Vernaya* occurred approximately 12 Ma ago. At this time, what caused the two genera to diverge caught our attention. During the 14–12 Ma, the Qinghai-Tibetan Plateau (QTP) is thought to have experienced a rapid uplift (Coleman and Hodges 1995; Dettman et al. 2003). Moreover, the uplift of the Sanjiang area in the southern section of the Hengduan Mountains is believed to have occurred at 25–17 Ma and 13–8 Ma (Zhong and Ding 1996). These events have created complex land conditions and diversified climate evolution (Wu et al. 2001), leading to the differentiation and evolution of specific species. Therefore, based on these findings, we speculate that the two climbing genera, *Micromys* and *Vernaya* may have been closely related in the early stage; later, due to the uplift of the QTP and the change in climate, the habitats of the two have changed and occupied different ecological niches, which led to their divergence. Moreover, should *Vernaya* and *Micromys* be subject to distinct selective pressures within their mitochondrial genomes, the discovery of adaptive sites that fuel speciation is a thrilling prospect that beckons us to delve deeper into this evolutionary enigma.

## ﻿Conclusions

We sequenced the mitochondrial genomes of four species of *Vernaya* and found that there were 2 D-loops in *V.meiguites.* There are relatively few relevant data and studies, and more research is needed to reveal the specific mechanisms and biological significance behind this phenomenon. In addition, we determined the phylogenetic relationships of Murinae based on the vast majority of its mtGenome data for the first time. We revealed many new details concerning the overall phylogenetic structure of Murinae and described its evolutionary history. We also propose a new tribe namely Vernayini Liu, Zhao, Liu and Chen, trib. nov. We believe that it is necessary to combine morphological and molecular data (especially from a genome-wide perspective) to determine the phylogenetic position in Murinae of some tribes (Vandeleurini, Pithecheirini, Phloeomyini, etc.) with an uncertain position, and the taxonomic status of Murinae*incertae sedis* (*Nilopegamys* and *Hadromys*). Further sampling and research are necessary to analyze the origin, evolution and extinction of various tribes of Murinae and the adaptation mechanisms involved.

## Supplementary Material

XML Treatment for Vernayini

## ﻿Additional information

### Author ORCIDs

Shuang Liu https://orcid.org/0009-0001-4080-1115

Songping Zhao https://orcid.org/0009-0002-8895-6439

### Data availability

The sequence and annotation of the mtGenomes of *Vernayafulva*, *Vernayaforamena*, *Vernayanushanensis*, and *Vernayameiguites* were submitted to the NCBI. The accession numbers respectively in GenBank are OR085222 (https://www.ncbi.nlm.nih.gov/nuccore/OR085222), OR085220 (https://www.ncbi.nlm.nih.gov/nuccore/OR085220), OR085221 (https://www.ncbi.nlm.nih.gov/nuccore/OR085221), OR085219 (https://www.ncbi.nlm.nih.gov/nuccore/OR085219).

## Supplementary materials

Supplementary material 1 Bayesian and Maximum likelihood trees for Murinae and close-relative lineages based on the mtGenome and the accession numbers of mitochondrial sequences for the trees Data type docx https://binary.pensoft.net/file/1291834This dataset is made available under the Open Database License (http://opendatacommons.org/licenses/odbl/1.0/). The Open Database License (ODbL) is a license agreement intended to allow users to freely share, modify, and use this Dataset while maintaining this same freedom for others, provided that the original source and author(s) are credited.Shuang Liu, Songping Zhao, Jing Wang, Changkun Fu, Xuming Wang, Shaoying Liu, Shunde Chen
