# Supplementary material for: ﻿New insights into the mitogenomic phylogeny and evolutionary history of Murinae (Rodentia, Muridae) with the description of a new tribe
Source: Zookeys. 2025 Mar 27;1233:55–74. doi: 10.3897/zookeys.1233.140676 (PMC11969158; doi:10.3897/zookeys.1233.140676)
Supplement: Supplementary material 1 — Bayesian and Maximum likelihood trees for Murinae and close-relative lineages based on the mtGenome and the accession numbers of mitochondrial sequences for the trees [file zookeys-1233-055_article-140676__-s001.docx]

**Supplementary Information**


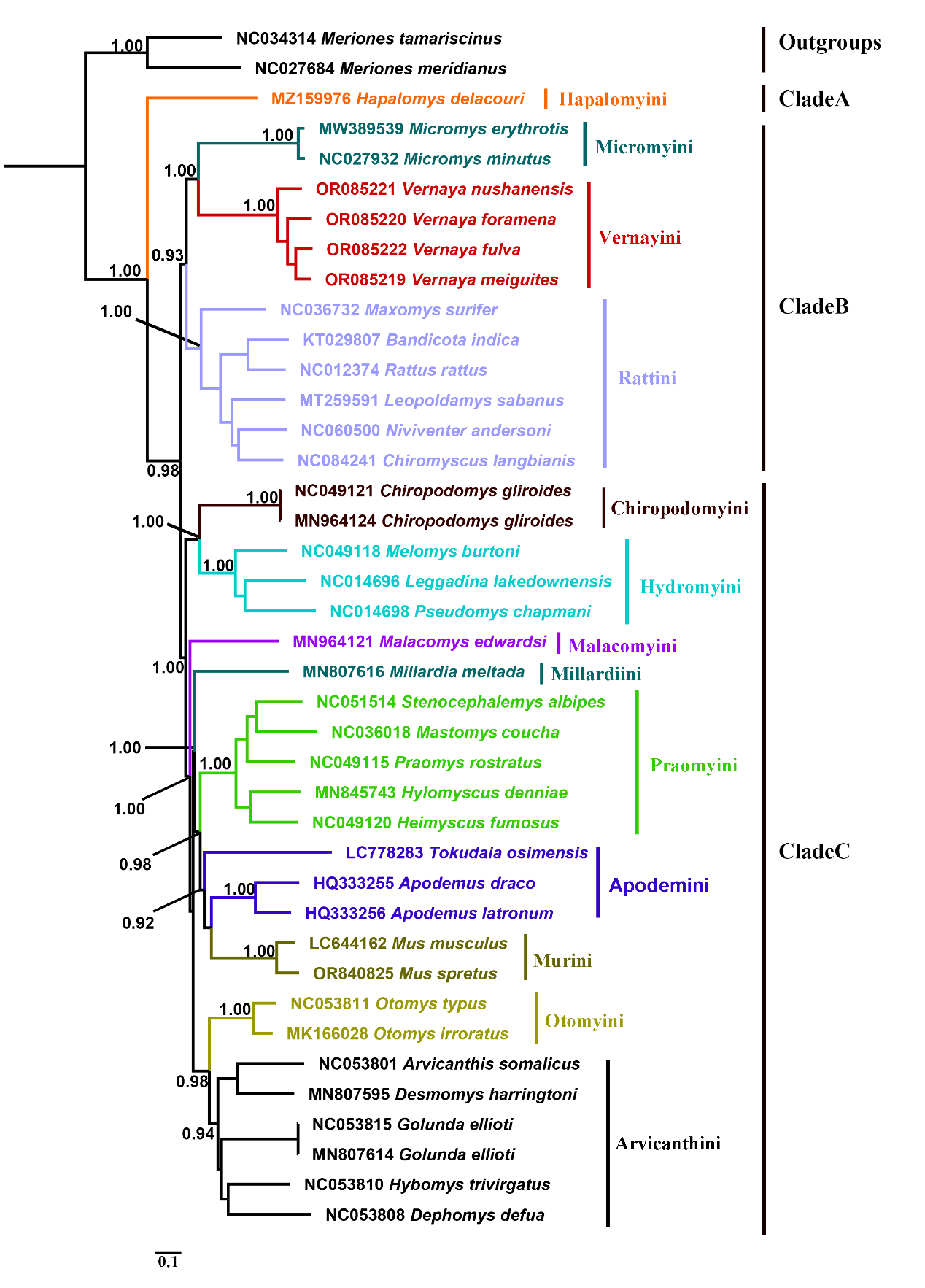


**Fig. S1.** Bayesian phylogenetic analyses based on mtGenome. The numbers above the branches refer to Bayesian posterior probabilities (PP).


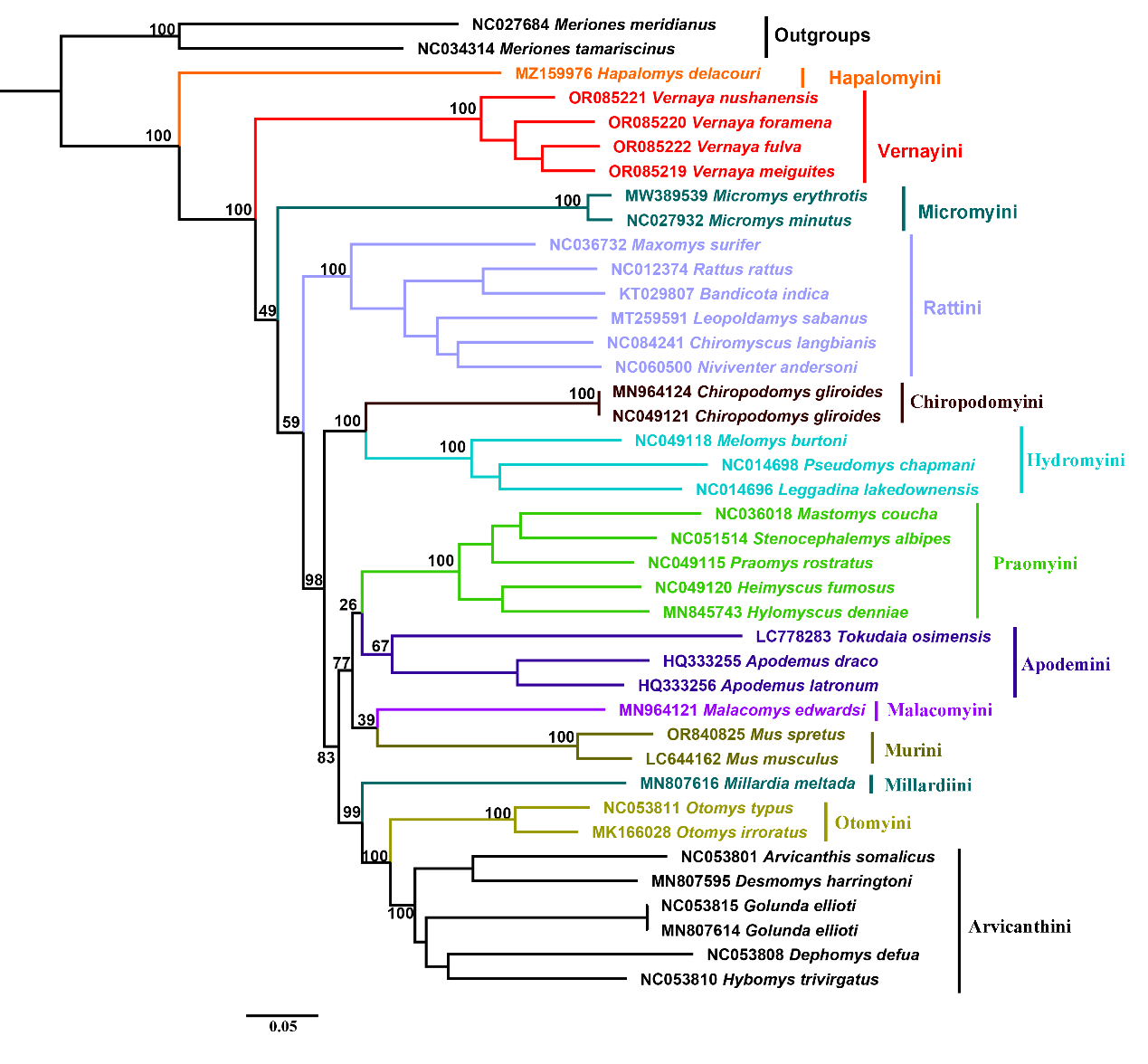


**Fig. S2.** Maximum-likelihood tree for Murinae and close-relative lineages based on the mtGenome. 1000 bootstraps were applied. Numbers at nodes represent ML bootstrap support values.

**Table S1.** Accession numbers of mitochondrial sequences of Murinae species used in Fig. S1 and Fig. S2.

| Tribes | species | Complete mitochondrial sequence |
| --- | --- | --- |
| Outgroups | *Meriones tamariscinus* | NC034314 |
|  | *Meriones meridianus* | NC027684 |
| Vernayini | *Vernaya foramena* | OR085220 |
|  | *Vernaya fulva* | OR085222 |
|  | *Vernaya meiguites* | OR085219 |
|  | *Vernaya nushanensis* | OR085221 |
| Malacomyini | *Malacomys edwardsi* | MN964121 |
| Rattini | *Bandicota indica* | KT029807 |
|  | *Niviventer andersoni* | NC060500 |
|  | *Rattus rattus* | NC012374 |
|  | *Chiromyscus langbianis* | NC084241 |
|  | *Leopoldamys sabanus* | MT259591 |
|  | *Maxomys surifer* | NC036732 |
| Micromyini | *Micromys minutus* | NC027932 |
|  | *Micromys erythrotis* | MW389539 |
| Praomyini | *Stenocephalemys albipes* | NC051514 |
|  | *Heimyscus fumosus* | NC049120 |
|  | *Hylomyscus denniae* | MN845743 |
|  | *Mastomys coucha* | NC036018 |
|  | *Praomys rostratus* | NC049115 |
| Millardiini | *Millardia meltada* | MN807616 |
| Chiropodomyini | *Chiropodomys gliroides* | NC049121 |
|  | *Chiropodomys gliroides* | MN964124 |
| Hydromyini | *Melomys burtoni* | NC049118 |
|  | *Leggadina lakedownensis* | NC014696 |
|  | *Pseudomys chapmani* | NC014698 |
| Apodemini | *Apodemus draco* | HQ333255 |
|  | *Apodemus latronum* | HQ333256 |
|  | *Tokudaia osimensis* | LC778283 |
| Otomyini | *Otomys typus* | NC053811 |
|  | *Otomys irroratus* | MK166028 |
| Arvicanthini | *Golunda ellioti* | NC053815 |
|  | *Golunda ellioti* | MN807614 |
|  | *Desmomys harringtoni* | MN807595 |
|  | *Arvicanthis somalicus* | NC053801 |
|  | *Dephomys defua* | NC053808 |
|  | *Hybomys trivirgatus* | NC053810 |
| Hapalomyini | *Hapalomys delacouri* | MZ159976 |
| Murini | *Mus spretus* | OR840825 |
|  | *Mus musculus* | LC644162 |
